# The mode of delivery after operative fixation of pelvic ring fractures–a retrospective observational study

**DOI:** 10.1007/s00068-024-02618-4

**Published:** 2024-08-10

**Authors:** Anna H. M. Mennen, Jelle J. Posthuma, Eline M. Kooijman, Marjolijn D. Trietsch, Eefje N. De Vries, Frank W. Bloemers, J. Carel Goslings, Daphne Van Embden

**Affiliations:** 1 Department of Surgery, Amsterdam UMC Location University of Amsterdam, Amsterdam, Netherlands; 2 Department of Surgery, Flevoziekenhuis, Almere, Netherlands; 3 Department of Surgery, Amsterdam UMC Location Vrije Universiteit Amsterdam, Amsterdam, Netherlands; 4 Department of Obstetrics and Gynaecology, Ikazia Hospital, Rotterdam, Netherlands; 5 Department of Surgery, Tjongerschans Hospital, Heerenveen, Netherlands; 6 Department of Surgery, OLVG Hospital, Amsterdam, Netherlands; 7 Department of Obstetrics and Gynaecology, Leiden University Medical Center, Leiden, Netherlands

**Keywords:** Pelvic fracture, Retained hardware, Mode of delivery, Surgical fixation, Vaginal delivery, Caesarean section

## Abstract

**Purpose:**

The purpose of this study is to investigate whether retained hardware after surgical treatment for a pelvic fracture prior to pregnancy affects the choice of delivery method. The study aims to provide insights into the rates of vaginal delivery and caesarean sections, understanding whether the mode of delivery was influenced by patient preference or the recommendations of obstetricians or surgeons, and examining the rate of complications during delivery and postpartum.

**Methods:**

All women of childbearing age who underwent surgical fixation for a pelvic ring fracture between 1994 and 2021 were identified. A questionnaire was sent about their possible pregnancies and deliveries. Of the included patients, surgical data were collected and the fracture patterns were retrospectively classified. Follow-up was a minimum of 36 months.

**Results:**

A total of 168 women with a pelvic fracture were identified, of whom 13 had a pregnancy after surgical stabilization. Eleven women had combined anterior and posterior fracture patterns and two had isolated sacral fractures. Four women underwent combined anterior and posterior fixation, the others either anterior or posterior fixation. Seven women had a total of 11 vaginal deliveries, and 6 women had 6 caesarean sections. The decision for vaginal delivery was often the wish of the mother (n = 4, 57%) while the decision to opt for caesarean section was made by the surgeon or obstetrician (n = 5, 83%). One woman in the vaginal delivery group suffered a postpartum complication possibly related to her retained pelvic hardware.

**Conclusion:**

Women with retained hardware after pelvic ring fixation can have successful vaginal deliveries. Complications during labor or postpartum are rare. The rate of primary caesarean sections is high (46%) and is probably influenced by physician bias. Future research should focus on tools that can predict labor outcomes in this specific population, and larger multicenter studies are needed.

**Level of evidence:**

Level III.

## Introduction

The incidence of pelvic ring fractures is approximately 20 per 100,000 persons per year in Western countries [1]. This injury predominantly occurs in adult male patients after high-energy trauma, and in elderly women with pre-existing osteoporosis after low energy trauma [2]. However, there is a significant group of young women who suffer a pelvic ring fracture each year, with recent literature reporting an incidence of around 15 pelvic ring fractures per 100.000 per year in young adult women [3]. Given that high-energy trauma in adults often leads to more severe and unstable types of pelvic fractures, many of these women undergo surgical treatment for their injury. This surgical treatment can range from minimally invasive percutaneous techniques to more open invasive techniques, both using osteosynthesis material spanning over the pubic symphysis and/or sacroiliac joints [4].

In the medical field, there is a strong belief that widening of the symphysis and sacroiliac joint during delivery is necessary for a successful vaginal birth [5]. This widening is often not possible in patients who have retained hardware following pelvic ring fixation. Additionally, injury and scarring of the pelvic floor muscles caused by the initial trauma and subsequent fracture patterns can affect the widening capacity of the pelvis during vaginal delivery. Lateral compression (LC) type injuries can cause relaxation of the pelvic floor, and anteriorposterior compression (APC) or vertical shear (VS) injuries can cause tension and disruption to the pelvic floor [6]. The prevalence of genitourinary and reproductive complaints in women who have sustained a pelvic fracture highlights this issue [6]. Despite this, the exact impact of pelvic ring fixation on childbirth outcomes has been sporadically described. To date, only seven studies have reported data on delivery after pelvic fracture fixation [7–13]. One of these studies is a case report with a low level of evidence [13], and four of these studies group the data of patients who underwent pelvic fracture fixation with those treated conservatively [9–12]. While in theory it is plausible that a history of pelvic fracture could interfere with the possibility to safely delivering vaginally due to residual pelvic asymmetry and changes in the diameter of the pelvic inlet and outlet, in general, those patients would be surgically treated. The issue of retained pelvic hardware during delivery has been largely overlooked in research. Hsu et al. described the birth outcomes of a cohort of 21 women who underwent pelvic ring fixation; however, 16 of these women had their hardware removed prior to delivery [8]. Since removal of pelvic hardware is not standard practice in Europe, the concern lies with patients who have retained hardware during delivery spanning over the SI-joint and/or symphysis. Parry et al. conducted the only other study investigating if a history of pelvic fracture fixation should be an indication for cesarean section, providing birth data of 10 women [7]. This study describes a high percentages of caesarean section of 40%. However, considering that caesarean section rates in Northern America (32%) are relatively high compared to Western Europe (26%) and the Netherlands (17%), it is unclear if these numbers reflect the current practice in our region [14, 15]. This lack of evidence regarding the necessity for primary caesarean section after fixation of the pelvic ring leads to challenging decisions in clinical practice for both physicians and patients.

The vaginal birth rate in The Netherlands is much higher compared to other high-income countries like the United States of America (75% vs 61%, respectively) [15, 16]. Despite this unique preference of Dutch women for vaginal delivery, we hypothesize that women with retained pelvic hardware following surgical fixation for pelvic ring fractures are more likely to experience a higher rate of caesarean sections compared to the general population, primarily due to physician recommendations rather than patient preference. Therefore, the aim of this study was to give insight into (1) the rate of vaginal delivery and caesarean sections in Dutch women who had undergone surgical fixation for their pelvic fracture in the past, (2) if the mode of delivery was based on patient preference vs. obstetrician or surgeon preference, and (3) the rate of complications during delivery and postpartum.

## Methods

### Study design

This study is reported in accordance with the Strengthening the Reporting of Observational Studies in Epidemiology (STROBE) criteria and checklist. This retrospective cohort study was conducted at two Level 1 trauma centers (Amsterdam UMC location AMC and location VUmc). This study aimed to investigate the outcomes of women who underwent surgical fixation of a pelvic ring fracture and subsequent pregnancy and delivery.

Women who underwent surgical fixation of a pelvic ring fracture between 1994 and 2021 were identified through electronic medical records. The inclusion criteria were:

- Women of childbearing age (15–40 years) at the time of injury.
- Women who had a pelvic ring fracture treated with surgical fixation
- Women who had at least one successful pregnancy (defined as carrying a pregnancy to full term) following the pelvic ring fixation.

Exclusion criteria for further analysis were:

- Known fertility problems.
- Patients who had not actively tried to become pregnant.
- Patients who did not have a successful pregnancy

All women who were eligible for inclusion were approached for participation in the study between January 2021 and February 2022, and were sent a questionnaire. The questionnaire used in this study was not a validated instrument. We opted to use an unvalidated questionnaire because, to our knowledge, no validated questionnaire exists that addresses the specific topics of interest in this study. The questionnaire was developed to cover key areas including demographics, medical history, and delivery-related information relevant to women who underwent surgical fixation of a pelvic ring fracture. While the lack of validation is a limitation, the questionnaire was designed to ensure comprehensive coverage of relevant topics based on expert input and clinical relevance. Details about the questionnaire can be found in Appendix 1 Exclusion criteria for further analysis were known fertility problems, patients who had not actively tried to become pregnant or had no successful pregnancy (e.g. pregnancy was not carried to full term).

### Sample size

We did not perform a formal power analysis due to the anticipated low sample size. Instead, we aimed to include all relevant patients possible to maximize the available data. This approach was chosen to ensure that the study would encompass the entire population of interest within the given timeframe and settings.

### Data collection

The data extracted from the electronic medical records included demographics such as age at the time of trauma, parity status (nulliparous or multiparous), history of cardio-pulmonary disease, diabetes, and smoking status. Injury characteristics were recorded, including the mechanism of injury (e.g., fall from height, motor vehicle accident), Injury Severity Score (ISS), and fracture classification according to the Young & Burgess classification for pelvic ring fractures and the Denis classification for isolated sacral fractures [17, 18]. Surgical data included the type of surgical fixation (e.g., SI-screws, symphysis plate, combined anterior and posterior fixation), date of surgery, and any subsequent hardware removal. Radiologic follow-up involved reviewing initial plain anteroposterior, inlet, and outlet radiographs and/or computed tomography scans of the pelvis to characterize the injury. All radiologic assessments and the retrospective classification of the injury were performed by two trained surgeons not involved in the patients’ care. Additionally, pregnancy and delivery outcomes were collected, including the mode of delivery (vaginal or caesarean), the number of successful pregnancies post-fixation, any complications during delivery (e.g., postpartum hemorrhage, vacuum extraction), and the decision-making process regarding the mode of delivery.

The primary outcome was the mode of delivery after fixation of the pelvic ring. Secondary outcomes were removal of osteosynthesis material prior to the pregnancy, interventions during delivery, secondary caesarean section, and complications during pregnancy and delivery.

### Data analysis

Quantitative variables were analyzed by grouping and comparing relevant data to examine the influence of retained pelvic hardware on delivery outcomes. Primary variables included the rates of vaginal delivery and caesarean sections, while secondary variables included delivery and postpartum complications, and fracture pattern classifications. Women were categorized by mode of delivery (vaginal or caesarean), type of pelvic fracture (according to the Young & Burgess classification), and type of surgical fixation (combined anterior and posterior hardware vs. anterior or posterior hardware alone). Due to the small sample size, only descriptive statistics were used to summarize the data. Women who were loss to follow-up were excluded from the study. This resulted in a complete dataset with no missing data, therefor there was no need for imputation or other methods to handle missing data.

### Ethical approval

This study was approved by the Medical Ethics Review Committee (METC) of the AMC (protocol number W20_084#20.112) and VUmc (protocol number 2020.352).

## Results

A total of 168 women with a pelvic fracture were identified, of whom 86 underwent surgical fixation. Of these 86 women, 66 were of childbearing age (15–40 years) at the time of injury and eligible for inclusion. Twelve women were lost to follow up due to a lack of contact information.

The questionnaire was sent to a total of 54 women, of which four women did not wish to participate in the study. The remaining 50 women all completed the questionnaire, resulting in a response rate of 92.6%. Thirty-six did not have a successful pregnancy after pelvic ring fixation and were excluded from further analysis; 13 because they choose to not have a child and 23 because they did not conceive. A total of 13 women had one or multiple successful pregnancies after pelvic ring fixation and were included for further analysis.

### Patient characteristics

Of these 13 women, the average age at trauma was 25 years old (12–32). Eleven women (85%) were nulliparous at the time. None of the women had a medical history of cardio- or pulmonary disease or diabetes. Only one woman was an active smoker. The pelvic ring fractures were the result of a fall from height in five (38%) patients and a high-velocity motor vehicle accident in eight patients (62%). An Injury Severity Score (ISS) of > 16 was seen in 9 patients (69%). Definitive surgical treatment was performed between 2001 and 2019. Median follow-up was 165 months (36–313).

### Pelvic fracture type

Combined anterior and posterior injuries were seen in the majority of the patients (n = 11, 85%). Lateral compression (LC) fractures were observed in two cases (15%); one LC1 and one LC3 type fracture. Anterior–posterior compression (APC) fractures were seen in three cases (23%); two APC type 2 and one APC type 3. Vertical shear fractures were seen in two cases (15%) and other fracture types were observed in four (31%) cases. The other fracture types consisted of the following fracture patterns; one woman with an isolated sacral fracture (Denis type 1) and contralateral LC1 fracture, two women with isolated sacral fractures (Denis type 2) with contralateral LC1 fracture, and one woman with an isolated sacral fracture (Denis type 1) and contralateral LC2 fracture. Isolated sacral fractures without anterior involvement were observed in two cases (15%); one Denis type 2 and one Denis type 3 fracture. Two patients had open fractures; one patient with an APC type 2 and one patient with a vertical shear fracture.

### Surgical fixation

The majority of the patients (n = 7, 54%) were treated with SI-screws; 4 women with unilateral, and 3 with bilateral SI-screws. Combined anterior and posterior fixation was performed in 4 women (31%), 3 by SI-screw and symphysis plate and 1 patient had bilateral SI-screws and a supra-acetabular external fixation. Two women (15%) were treated by symphysis plate only.

With the exception of the removal of the external fixation in one woman 10 weeks post-surgery, no removal of hardware was performed prior to the pregnancies.

### Mode of delivery

All women had some form of retained hardware during either vaginal delivery or caesarean section. Overall, the 13 women gave birth to 17 babies in total (one twin birth). Eleven babies were born as the result of vaginal deliveries by 7 women, and 6 babies were born by means of planned caesarean section in 6 women. Both multiparous women had vaginal deliveries after surgical fixation. There were no other notable differences regarding patient characteristics between the two groups. For details of the two groups, see Table 1.

**Table 1:** Baseline characteristics

	Vaginal delivery (n = 7 females)	Caesarean section (n = 6 females)
Age at trauma (median (IQR range))	24.5 years (20–31)	25 years (12–32)
Gravidity and parity		
Before trauma		
G0P0	4 (57%)	5 (83%)
G1P0	1 (14%)	1 (17%)
G2P2	2 (29%)	
End of follow-up		
G1P1	2 (29%)	4 (67%)
G2P1	1 (14%)	2 (33%)
G2P2	2 (29%)	
G4P3	1 (14%)	
G4P4	1 (14%)	
Active smoker	1 (14%)^a^	0 (0%)
Medical history		
Diabetes	0 (0%)	0 (0%)
Pulmonary disease	0 (0%)	0 (0%)
Cardiovascular disease	0 (0%)	0 (0%)
Trauma mechanism		
Fall from height	2 (29%)	3 (50%)
Motor vehicle accident	5 (71%)	3 (50%)
Injury Severity Score		
< 16	2 (29%)	2 (33%)
> 16	5 (71%)	4 (67%)
Fracture type		
LC 1	1 (14%)	
LC 3		1 (17%)
APC 2		2 (34%)
APC 3	1 (14%)	
Vertical shear	1 (14%)	1 (17%)
Combined patterns	2 (29%)	2 (34%)
Denis 2	1 (14%)	
Denis 3	1 (14%)	
Fixation type		
SI-screw		
Unilateral	3 (43%)	1 (17%)
Bilateral	2 (29%)	1 (17%)
Symphysis plate	1 (14%)	1 (17%)
SI-screw + symphysis plate	1 (14%)	2 (33%)
Bilateral SI screws + supra-acetabular external fixation	0 (0%)	1 (17%)
Removal of hardware prior to delivery	0 (0%)	1 (17%)^b^

*SI* sacroiliac, *LC* lateral compression, *APC* anteroposterior compression

^a^Quit smoking during pregnancy

^b^Removal of supra-acetabular external fixation, SI-screws were kept in place

In the vaginal delivery group (n = 7), five women had combined anterior and posterior injuries, yet only one patient (14%) received combined anterior and posterior fixation by SI screw + symphysis plate. The other women with combined anterior and posterior injuries were treated by unilateral (n = 1, 14%) or bilateral (n = 2, 29%) SI-screw fixation, and one woman underwent plate osteosynthesis of the symphysis (14%). The two women (28%) with isolated sacral fractures both underwent unilateral SI-screw fixation.

The caesarean section group (n = 6) all had combined anterior and posterior injuries. Of these women, three received combined anterior–posterior fixation; two (33%) underwent unilateral SI-screw fixation combined with symphysis plating, and one (17%) patient was treated by bilateral SI-screws and supra-acetabular external fixation. Although the external fixator was removed 10 days after surgery, during follow-up imaging, ankylosing spondylitis involving the symphysis pubis could be seen, which resulted in an arthrodesis of this joint. The other women underwent unilateral (n = 1, 17%) or bilateral (n = 1, 17%) SI-screw fixation, or plate osteosynthesis of the symphysis (n = 1, 17%) (Table 1). Both women with open pelvic fractures underwent caesarean section. There was one woman who was treated by unilateral SI-screws for her isolated sacral fracture and contralateral LC1 fracture in the vaginal delivery group who suffered from postpartum hemorrhaging (9%). One woman who suffered a vertical shear fracture and was treated with unilateral SI-screws had vacuum extraction delivery due to failure to progress (9%). None of the women in the vaginal delivery group underwent a secondary caesarean section. In the caesarean section group, no peripartum complications were observed. In addition, one patient in the caesarean section group with an extensively open APC2 fracture treated with a unilateral SI-screw and external fixation had a planned delivery by caesarean section at 36 weeks due to concerns about her soft-tissue issues in the anorectal region (< 1 year after her sustained injury). Post-partum she developed a symptomatic widening of the SI-joint on the contralateral side, for which she was treated by an additional SI-screw (see Table 2).

**Table 2:** Complications and decision for mode of delivery

	Vaginal delivery (n = 11 deliveries)	Caesarean section (n = 6 deliveries)
Peripartum and postpartum complications		
Postpartum haemorrhage	1 (9%)	0 (0%)
Foetal distress	0 (0%)	0 (0%)
Assisted delivery (ventouse)	1 (9%)	0 (0%)
Emergency caesarean section	0 (0%)	0 (0%)
Decision for mode of delivery		
Patient	4 (36%)	0 (0%)
Surgeon	1 (9%)	3 (50%)
Obstetrician	0 (0%)	2 (33%)
Shared decision making	2 (18%)	1 (17%)

The decision to opt for vaginal delivery was often the wish of the mother (n = 4, 57%). In contrast, the decision to opt for caesarean section was mostly made by the surgeon or obstetrician (n = 5, 83%). It should be noted that one of these women was advised to deliver by caesarean section because of soft-tissue issues in the anorectal region caused by her open pelvic fracture instead of expected complications due to her retained hardware (see Table 2).

## Discussion

The results of this study suggested that safe vaginal delivery may be possible after pelvic fixation. The rate of primary caesarean sections is high and is probably influenced by physician bias. One woman even had a successful vaginal delivery with combined anterior and posterior fixation in situ.

Although caesarean section can be a life-saving intervention when medically indicated, the procedure can affect both women and babies. Several studies showed that women who underwent a caesarean section have an increased risk of obstetric complications and poorer outcomes for mother and baby [19–21] This should be taken into account given the increasing use of caesarean sections, particularly without medical indication [14]. The caesarean section rate in the Netherlands has risen from 5% in 1980 to 17% in 2020 [15]. Even though this number is rising, it is still below the average in Europe (26%) or Northern America (32%) [14]. In this cohort of 13 women, 46% underwent a caesarean section, which is much higher than the average caesarean section rate in our country. Previous studies show similar high rates of caesarean sections in women who suffered a pelvic fracture prior to their pregnancy (45%-60%) [11, 12]. This data was obtained from both operatively and non-operatively treated women, which shows that even without retained hardware there is a trend to advise primary caesarean section to these women.

To the best of our knowledge, only two papers published data on pregnancy outcomes among women who underwent operative treatment for a pelvic ring fracture. A case report by Goswami et al. described a patient who underwent anterior pelvic wiring and subsequently had a successful vaginal delivery [13]. The study of Vallier et al. reported six women who were operatively treated for their pelvic fracture and subsequently had a successful vaginal delivery without any complications [12]. In our study, one woman (9%) who delivered vaginally had a postpartum hemorrhage that did not need surgical intervention. The incidence of postpartum hemorrhage following a vaginal delivery in the general population varies between 1 and 12% [22, 23]. It seems unlikely that this complication is related to her having retained hardware. Labor complications that could potentially be related to cephalopelvic disproportion due to retained hardware or residual pelvic asymmetry are mainly failure to progress in labor, and less common shoulder dystocia and rupture of the uterus or laceration of the cervix [24, 25]. Nevertheless, only one of these potential complications was seen in our cohort, and none was described in the existing literature. This shows that safe vaginal delivery with retained hardware is possible, and suggests an overuse of caesarean sections even in women who were not surgically treated for their pelvic fracture.

One of the main contributors to the high rate of caesarean sections might be physician bias. After operative fixation of a pelvic fracture, patients have been advised by physicians to have a caesarean section in up to 21% of cases [6]. Similar numbers were seen in our cohort. The motivation for this advice is still grossly unknown. Part of this bias might be related to the belief that widening of the pubic symphysis and sacroiliac joints is a crucial process during a vaginal delivery, which would not be possible with retained hardware spanning these joints. In the past, this has been viewed as an indication for hardware removal in young women [26]. However, if the hardware has been in place for extended periods of time, it is highly questionable if there is any residual mobility left in the pelvic joint due to sclerosis. In addition, since safe vaginal delivery is possible with the hardware in place, this secondary surgery seems unnecessary. Another concern that might contribute to physician bias is the possibly reduced size of the pelvic outlet due to residual dislocation. The fetal-pelvic index is a measurement of the maternal pelvis size related to the size of the fetus, and was shown to be reliable in predicting fetal-pelvic disproportion and the likelihood of (un)successful vaginal delivery [27–29]. This tool could be potentially be useful in the obstetric care of women with a history of a pelvic ring fracture and a high degree of residual pelvic asymmetry or displacement. However, this tool has not been validated specifically for this group of patients, so future research is warranted.

The general preference of Dutch women for vaginal delivery as opposed to planned caesarean section allows for shared decision making and so called ‘trial of labor’, instead of physicians’ advice to perform a planned caesarean section. In such cases, patients should be advised to deliver under medical care with access to surgical intervention (no home delivery or delivery at a midwife-led center without access to operating theater).

### Strengths and limitations

Although our study is limited by the small size of our cohort, it is the first to focus primarily on women who gave birth with retained hardware. Due to the limited sample size, we cannot conclusively determine safety of vaginal delivery based on this study alone, but we can demonstrate that it is feasible. The questionnaire was as concise as possible but there might be a recall bias since some of the participants received the questionnaire several years after they gave birth. Due to the long inclusion period, the quality and type of treatment provided to these women might have changed over time, which could have influenced the outcomes. Furthermore, if the patient did not give birth in the Amsterdam UMC, the data on labor, postpartum complications and the indication for caesarean section was only based on the information provided by the women, which might also have caused some bias. Additionally, the generalizability of our findings may be limited due to the specific population studied, which primarily includes Dutch women treated at a single medical center. This may not fully represent the broader population of women with pelvic fractures and retained hardware globally or in other healthcare settings.

## Conclusion

Our findings suggest that vaginal delivery is possible for women with retained hardware after pelvic ring fixation, even with combined anterior and posterior retained hardware. However, the high rate of primary caesarean sections observed in our cohort highlights the potential influence of physician bias and underscores the need for shared decision-making in obstetric care. Future research should focus on validating tools that can predict labor outcomes in this specific population, such as the fetal-pelvic index, and on understanding the underlying reasons for the high rate of caesarean sections. Larger, multicenter studies are needed to confirm these findings and improve the generalizability of the results.

## Data Availability

No datasets were generated or analysed during the current study.

## Appendix 1

Questionnaire the mode of delivery after operative fixation of pelvic ring fractures

Demographic information

- 1. Date of birth (dd-mm-yyyy):
- 2. Gender: male/female.
- 3. Do you have a medical history of any of the following diseases?:
  o Diabetes
  o Heart or vascular disease
  o Pulmonary disease
  o None
- 4. Do you smoke?
  o No
  o Yes, number of cigarettes per day: _______
- 5. Have you smoked in the past?
  o No
  o Yes, from (year) ______ to (year) ______
- 6. How did you sustain the fracture?
  o Work
  o Traffic
  o Home
  o Sports
  o Fall from height
  o Other, namely: _______

Treatment information

- 7. Did you have any problems with the surgical wounds after the operation?:
  o Yes
  o No (you may skip the next question)
  o Not applicable, I was not operated on
- 8. If yes, what was the problem?:
  o Wound was not closed after 2 weeks
  o Infection, treated with antibiotics
  o Infection, requiring hospital admission and/or surgery
  o Other, namely: _______

Pregnancy and childbirth

9. Were you actively trying to conceive after the operation?:
  o Yes
  o No (skip rest of the questionnaire)
10. Have you been pregnant after the pelvic surgery?
  o Yes
  o No
11. If yes, how many times have you been pregnant? _______
12. How many children were born from these pregnancies?
13. Were there any complications during the pregnancy?
  o Yes
  o No
14. If yes, what complications occurred?
15. How did you give birth?
  o Vaginal delivery
  o Caesarean section
  o Other, namely: _______
16. Were there any complications during the delivery?
  o Yes
  o No
17. If yes, what complication?
18. If you gave birth by caesarean section, how was the decision for a caesarean made?
  o On the advice of the gynaecologist/midwife
  o At my own request
  o In consultation with the gynaecologist
  o Other, namely: _______
